# Optimized design and performance evaluation of long-pressure-short-extraction ventilation and dust removal system based on the Coanda effect

**DOI:** 10.3389/frai.2025.1565889

**Published:** 2025-04-03

**Authors:** Xinguo Wang, Jinbo Zhao, Yufu Li, Zhibin Li

**Affiliations:** ^1^Shangwan Coal Mine, Ordos, China; ^2^CCTEG Xi’an Research Institute Co. Ltd., Xi’an, China

**Keywords:** mine ventilation, Coandă effect, dust control, CNN-LSTM, pressure-to-suction ratio

## Abstract

Mine ventilation and dust control systems are crucial for ensuring occupational safety and health during underground mining operations. Traditional long-pressure short-suction systems face challenges such as inefficient airflow organization, formation of vortex dead zones, high energy consumption, and inadequate adaptability to dynamic conditions in mining faces. This study addresses these limitations by proposing an optimized long-pressure short-suction ventilation and dust control system leveraging the Coandă effect. Through numerical simulations, experimental validation, and machine learning techniques, the study develops a comprehensive system to enhance dust control performance. The Coandă effect was employed to optimize the structural design of ventilation ducts, ensuring airflow attachment to tunnel surfaces, reducing dust dispersion, and achieving high-efficiency airflow with lower power consumption. The key parameters optimized include the spacing between the air supply and exhaust ducts, the pressure-to-suction ratio, and the height of the ventilation duct. The optimal pressure-to-suction ratio was found to be 2:3, which minimizes dust concentration at both the mining machine and downstream locations. Numerical simulations and experimental results demonstrated that the optimized system achieved dust concentration reductions of up to 84.12% in high initial dust conditions (800 mg/m^3^). These findings provide a solid foundation for intelligent and energy-efficient ventilation and dust control in mining operations, ensuring both safety and energy savings.

## 1 Introduction

Ventilation and dust removal systems are crucial in underground mining operations, serving as a core means of ensuring occupational safety and health for workers (Yu et al., 2025a; Nie et al., 2024a; Nie et al., 2024b). In fully mechanized heading faces, high concentrations of dust and harmful gases not only pose safety risks but also reduce productivity (Yu et al., 2025b; Dey et al., 2021). These challenges are particularly severe in regions with high mining intensity, where dust generation is continuous and localized, often exceeding permissible concentration levels. The inability to effectively control dust dispersion contributes to poor air quality, which increases the risk of respiratory diseases and accidents in underground operations. Studies have shown that traditional long-pressure-short-extraction ventilation and dust removal systems have significant shortcomings. Due to unreasonable airflow organization, vortexes and dead zones are likely to form, leading to low dust removal efficiency in local areas. Moreover, maintaining sufficient ventilation volume typically requires high-power fans, resulting in significantly increased energy consumption. This issue is particularly prominent during long-distance airflow transportation or under complex operational conditions (Cai et al., 2021; Liu et al., 2022). Traditional systems also lack the ability to dynamically respond to the varying needs of fully mechanized heading faces, as airflow volume and pressure cannot adjust in real time based on changes in dust concentration and airflow velocity, further limiting system efficiency (Lu Y. et al., 2022; Zhou et al., 2022).

To address these issues, numerous researchers have conducted in-depth studies. Lu X. et al. (2022) investigated the influence of different cutting orientations of roadheaders on the diffusion of disorganized dust and found that the cutting orientation significantly affects dust concentration distribution. High-concentration dust exhibits an asymmetric linear decreasing characteristic, with the minimum wind speed zone migrating with changes in cutting orientation. Lu X. et al. (2022) proposed a “blocking-sealing” dust removal method, which adjusts the distribution of airflow energy by setting dust-blocking plates and utilizes negative pressure to effectively control high-concentration dust zones. When the suction volume was 600 m^3^/min, the high-concentration dust zone nearly disappeared, and the dust concentration in the roadway was reduced to below 172 mg/m^3^ with an air volume ratio of 1:1.5. Lu X. et al. (2022) developed a spray device based on the synergistic action of wind and spray, optimizing nozzle parameters through experiments and CFD simulations. In practical applications, this device reduced the total dust concentration at the driver’s position to 104.2 mg/m^3^ and respirable dust to 68.3 mg/m^3^, achieving dust removal efficiencies of 88.31 and 83.45%, respectively.

Dust concentration and particle size distribution characteristics have also become key research areas. Lu et al. (2022) revealed the dust concentration distribution patterns on the leeward side of the New An Coal Mine’s 3,401 fully mechanized mining face. Within a 23-meter range on the leeward side of the front drum center, dust concentrations reached 1,220–2,620 mg/m^3^. The Coanda effect, as an important phenomenon in fluid mechanics, has wide applications in optimizing ventilation duct designs. Yu et al. (2024) simulated the diffusion characteristics of cutting dust using the MRF method, demonstrating that the Coanda effect can significantly reduce dust dispersion and achieve more uniform airflow. A high-concentration dust belt approximately 10 meters in length was formed on the leeward side of the drum, closely matching real-world conditions. Optimized structures based on the Coanda effect can effectively improve airflow organization by ensuring airflow attachment along the tunnel wall, thereby reducing dust dispersion while lowering wind resistance and energy consumption. In fully mechanized mining faces, the optimized ventilation system can achieve efficient airflow at lower fan power, significantly enhancing system performance.

With the development of intelligent technologies, machine learning-based intelligent ventilation systems have become a research hotspot. Semin and Kormshchikov (2024) suggested that artificial intelligence (AI) technologies can effectively improve monitoring and control capabilities in complex ventilation networks, enabling rapid calculation of ventilation parameters and fault diagnosis, especially during emergencies such as underground explosions and fires. Wang et al. (2024) optimized airflow distribution in mine ventilation to reduce energy consumption. They developed a nonlinear optimization model, applied the minimum spanning tree method, and used a modified sooty tern optimization algorithm (mSTOA). The results showed a 35.06% reduction in energy consumption while maintaining ventilation constraints, improving mine safety and production. Hati (2022) studied intelligent airflow perception in metal mines using AI methods. They proposed a CNN-LSTM-based model to predict airflow at undisclosed locations using partial data from monitoring points. The model showed an average deviation of less than 5% between predicted and actual airflow parameters. This approach enhances ventilation safety, improves productivity, and reduces energy consumption. Furthermore, combining CNN-LSTM models allows for precise prediction of ventilation parameters. CNN extracts spatial features, while LSTM processes time-series data; the combination effectively captures dynamic relationships, supporting intelligent control of ventilation systems.

**Figure 1:**
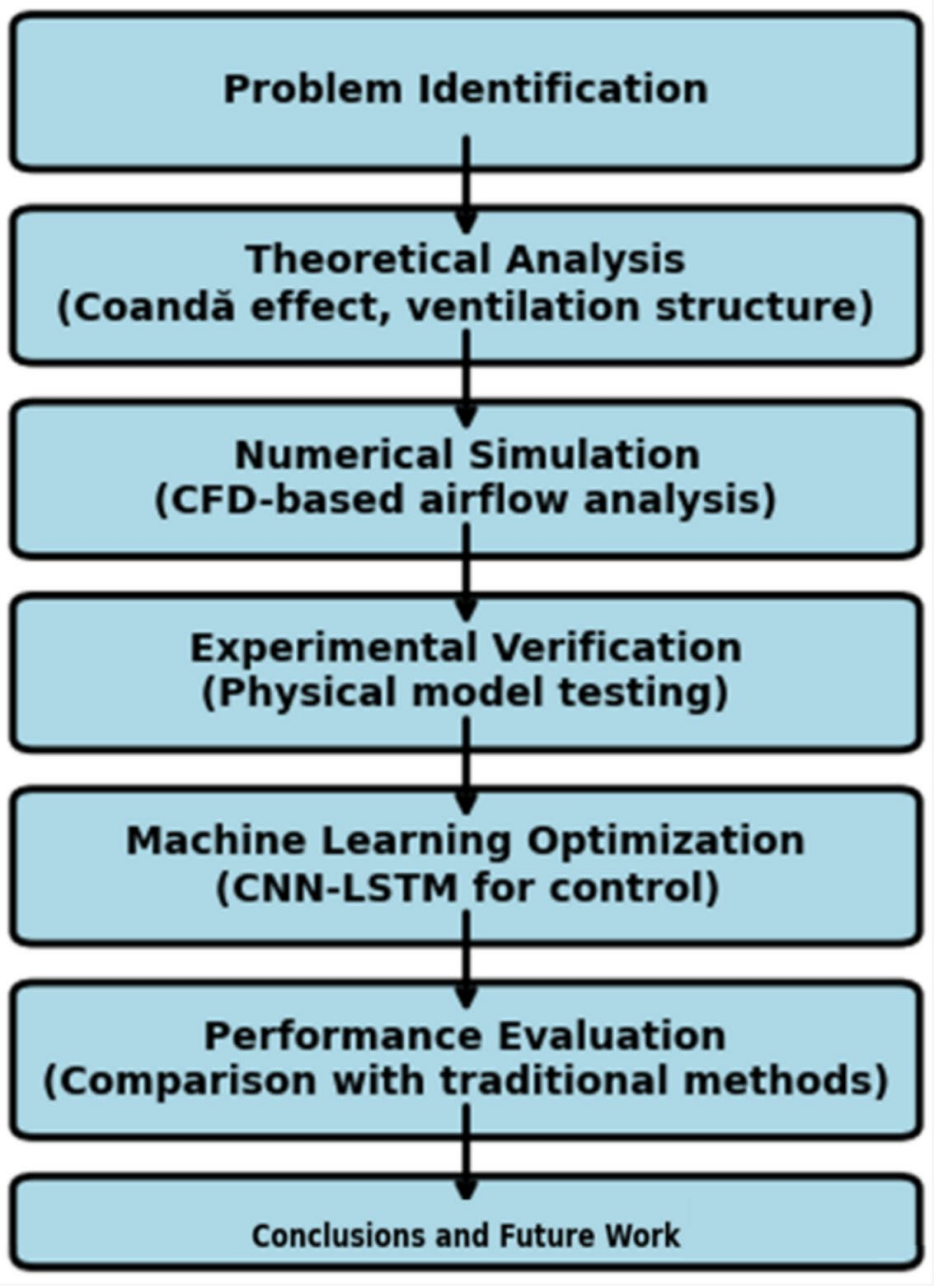
Research workflow.

The optimized system based on the Coanda effect, combined with the CNN-LSTM intelligent control model, demonstrates significant innovation in the field of ventilation and dust control, as shown in Figure 1. The main objectives of this research are to optimize the long-pressure-short-extraction ventilation and dust control system to enhance dust removal efficiency and reduce energy consumption. Through numerical simulations, experimental validation, and machine learning techniques, this study develops a comprehensive system to improve the performance of ventilation and dust removal in underground mining operations. The system uses the Coanda effect to ensure efficient airflow organization, effectively reducing dust dispersion while lowering wind resistance and energy consumption. Additionally, the CNN-LSTM model is employed to enable real-time intelligent control of ventilation system parameters, predicting and optimizing dust concentration levels with higher accuracy and adaptability. The expected outcomes include a significant reduction in dust concentrations, increased system efficiency, and a practical solution for dynamic adjustments to the ever-changing conditions of mechanized mining faces. This innovative approach aims to provide valuable insights into the development of intelligent dust control solutions, ensuring both occupational safety and energy-efficient operations in underground mining environments.

## 2 Theoretical basis

### 2.1 Optimization of ventilation and dust removal system structure based on the Coanda effect

**Figure 2:**
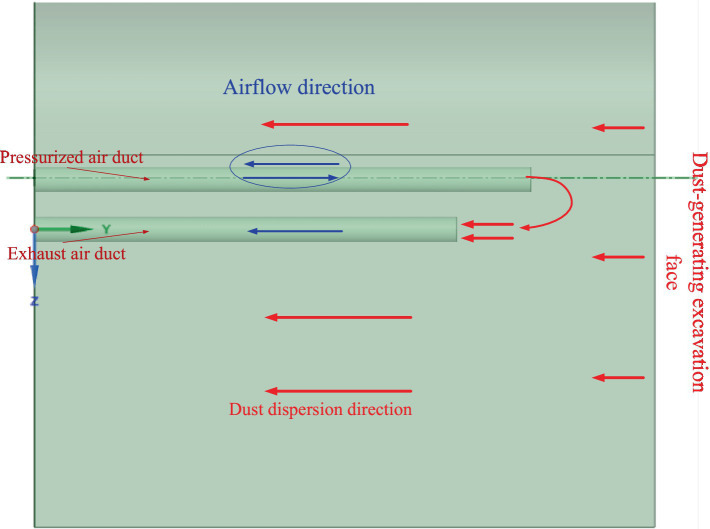
Traditional long-pressure-short-extraction ventilation duct structure.

Ventilation ducts are the core components of ventilation and dust removal systems. The traditional long-pressure-short-extraction ventilation duct structure is shown in Figure 2. Due to the unreasonable arrangement of ventilation ducts and fan positions, the airflow organization often becomes complex, leading to vortexes or dead zones. These phenomena prevent the effective removal of dust and harmful gases, resulting in low dust removal efficiency in localized areas (Lu et al., 2022; Zheng et al., 2023; Han et al., 2024). To maintain sufficient ventilation, traditional systems typically require high-power fans, which significantly increase energy consumption. This issue is particularly pronounced in scenarios involving long-distance airflow transportation or complex working conditions. Furthermore, traditional ventilation systems lack adaptive regulation capabilities to respond to dynamic environmental changes, making them incapable of real-time adjustments based on the evolving ventilation needs of fully mechanized heading faces. Airflow volume and pressure are usually preset and cannot be dynamically adjusted according to real-time parameters such as dust concentration or airflow velocity, further reducing system efficiency.

**Figure 3:**
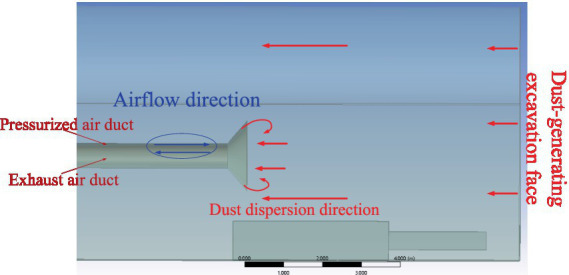
Optimized ventilation duct structure utilizing the Coanda effect.

The Coanda effect, a phenomenon in fluid mechanics, refers to the tendency of a fluid to adhere to a curved surface or obstacle and follow its contour. The optimized ventilation duct structure utilizing the Coanda effect is illustrated in Figure 3. In this design, the negative-pressure duct and the exhaust duct are arranged in a nested configuration, allowing the airflow within the negative-pressure duct to generate the Coanda effect at the exhaust duct outlet. In traditional systems, airflow often deviates from the intended path, whereas the optimized structure ensures that the airflow remains attached to the tunnel wall, significantly reducing the dispersion of airborne dust. As a result, the ventilation and dust removal system achieves efficient airflow at lower fan power, substantially improving overall performance (Hou et al., 2023). The Coanda effect significantly improves airflow organization by ensuring that the airflow adheres to the tunnel surface, which reduces dust dispersion and enhances airflow efficiency. Specifically, the Coanda effect ensures that the airflow remains attached to the tunnel wall at the exhaust duct outlet, preventing vortex formation and dead zones. This improves dust collection efficiency while reducing energy consumption. The proposed ventilation structure combines nested ducts and the Coanda effect, leading to significant improvements in system performance and dust removal.

### 2.2 CNN-LSTM model

#### 2.2.1 CNN

Convolutional Neural Network (CNN) is one of the most popular and widely used deep learning models in recent years. It consists of convolutional layers, pooling layers, and fully connected layers (Wang et al., 2021; Li et al., 2024). Compared to traditional neural networks, CNN replaces general matrix operations with convolutional operations, achieving significantly higher computational efficiency, as shown in Equation 1. The general form of convolutional operations is:


(1) s=x∗w

Where, **s** represents the feature map obtained after convolution, **x** is the input matrix, **w** is the weight matrix (i.e., the kernel function), and * denotes the convolution operator. Generally, as shown in Equation 2, the input for convolution operations is in a multi-dimensional form, so the weight matrix is also multi-dimensional. The number of convolution kernels determines the level of abstraction for feature extraction, and the kernel size can be adjusted according to the size of the input sequence data.


(2) $$s_{p,q}=\sum_{l=1}^{L}\sum_{m=1}^{M}i_{l,m}k_{p+l,q+m}$$

In the formula, *sp*;*q* represents an element of matrix s, *il*;*m* is an element of the 2D input matrix I with dimensions L × M, and *kp* + *l*;*q* + *m* is an element of the 2D convolution kernel K.

CNN operates using sparse connections and shared weights to directly extract effective feature information from raw data through the combined operations of convolutional and pooling layers. This process allows CNN to automatically extract local features from the data and form complete feature vectors.

#### 2.2.2 LSTM

LSTM, as a variant of Recurrent Neural Networks (RNN), leverages gate units for logical control to manage data operations. It provides a solution to the issues of insufficient long-term sequence information memory, gradient vanishing, and gradient explosion that commonly occur in RNNs. LSTM is capable of retaining information from time steps significantly distant from the current time step, making it better suited for processing time series data with extended time spans (Jianping et al., 2021).

**Figure 4:**
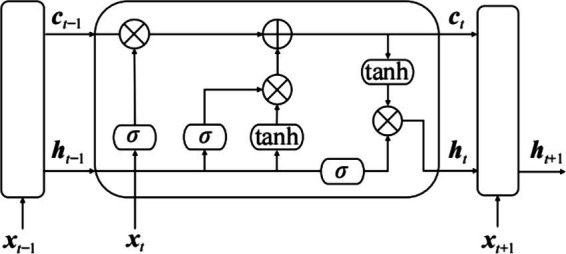
Structure of LSTM neural network unit.

The structure of each unit in an LSTM neural network is shown in Figure 3, which includes an input gate, a forget gate, an output gate, and a memory cell. The logical control of the memory cell determines how data is processed, effectively addressing the impact of weights on network training and allowing for better convergence (Kumari et al., 2021; Huang et al., 2022; Zhang et al., 2024). In Figure 4, *x_t_* represents the input value at the current time *t*, *h_t − 1_* is the output value of the hidden layer at the previous time step, *c_t_* is the state information at the current time t, and *σ* denotes the sigmoid function.

The forget gate is responsible for determining the extent to which the output information from the previous time step t − 1 should be retained. The output of the forget gate, ***f**t*, is calculated using ***x**t* and ***h**t* − 1 as shown in Equation 3, where ***f**t* ∈ [0; 1]. Its expression is as follows:


(3) $$f_{t}=\sigma \left(W_{f}\left[h_{t-1},x_{t}\right]+b_{f}\right)$$

In the equation: ***W***f represents the weight matrix of the forget gate, and ***b***f is the bias term of the forget gate. When the output of the forget gate is 0, it indicates that all output information from the previous time step is forgotten. Conversely, when the output is 1, all output information from the previous time step is fully retained.

The input gate, on the other hand, controls the output to allow only the useful information at the current time step to be input into the network, as shown in Equation 4.


(4) $${\tilde{f}}_{t}=\sigma \left(W_{i}\left[h_{t-1},x_{t}\right]+b_{i}\right)$$

In the equation, ***W***i represents the weight matrix of the input gate, and ***b***i is the bias term of the input gate.

The memory cell is used to compute the state information ***c**t* at the current time step t. The state information from the previous time step ***c**t* − 1 is multiplied by the output of the forget gate to retain information from the previous step. The state information $\tilde{c}$ at the current time step is multiplied by the output of the input gate to obtain the memory information for this time step. These two components are then combined to form the new state information ct. The computation process is as shown in Equations 5, 6:


(5) $${\tilde{c}}_{t}=\tanh\left(W_{c}\left[h_{t-1},x_{t}\right]+b_{c}\right)$$

(6) $$c_{t}=f_{t}c_{t-1}+{\tilde{f}}_{t}{\tilde{c}}_{t}$$

In Equations 5, 6, ***W***c represents the weight matrix of the memory cell, and ***b***c is the bias term of the memory cell. The output gate processes ct through a nonlinear function to obtain the output ***o**t* of the LSTM network:


(7) $$o_{t}=\sigma \left(W_{o}\left[h_{t-1},x_{t}\right]+b_{o}\right)$$

(8) $$h_{t}=o_{t}\tanh c_{t}$$

In Equations 7, 8, ***W***o represents the weight matrix of the output gate, and ***b***o is the bias term of the output gate.

#### 2.2.3 Optimization framework of CNN–LSTM for ventilation and dust removal system parameters

In this study, the CNN-LSTM hybrid model is proposed to optimize ventilation and dust removal system parameters. The LSTM model has limitations in handling multi-dimensional factors, while CNN excels in feature extraction but cannot capture temporal dependencies. By combining the strengths of both, the CNN-LSTM hybrid model is constructed. The framework uses convolutional layers to extract significant spatial features and LSTM layers to model time-dependent relationships, addressing the challenges in optimizing complex ventilation systems.

**Figure 5:**
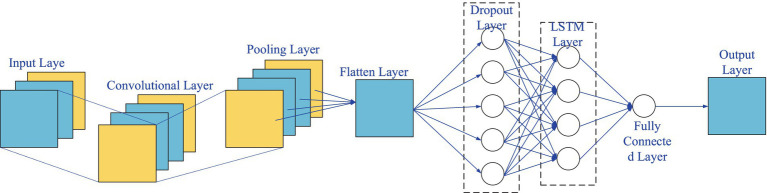
Structure of CNN–LSTM forecasting model.

The CNN-LSTM model includes several components: an input layer, convolutional layers for feature extraction, pooling layers for down-sampling, LSTM layers for temporal pattern modeling, and fully connected layers for output generation. The inclusion of Dropout layers helps mitigate overfitting by discarding certain network units during training, thereby improving model generalization. The Flatten layer ensures that data is appropriately formatted for the LSTM layers, as shown in Figure 5.

## 3 System design and optimization

### 3.1 Structural design of the long-pressure short-suction ventilation and dust removal system based on the Coandă effect

Using the 22,106 working face of Shangwan Coal Mine as the research object, the dimensions of the roadway and the dust removal devices were adjusted according to the actual conditions of the excavation working face. A simulated roadway measuring 50 m × 5.0 m × 4.0 m (length × width × height) was constructed to simulate and analyze the movement patterns and concentration distribution of dust particles. The main component dimensions of the model are listed in Table 1.

**Table 1:** Main component dimensions of the model.

Component name	Dimensions
Pressurized air duct	ϕ0.65 m
Exhaust duct	ϕ0.50 m
Pressurized air duct nozzle	ϕ1.8 m
Exhaust duct nozzle	ϕ1.5 m
Dust-generating surface	ϕ0.8 m
Roadheader	6.5 m × 2.0 m × 1.0 m (L × W × H)

**Figure 6:**
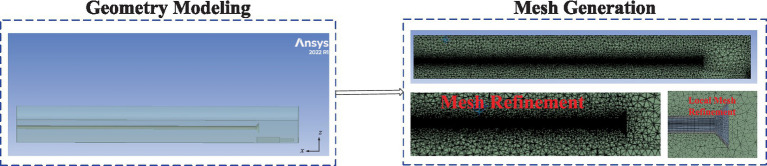
Optimized geometric model of the ventilation and dust removal system.

**Figure 7:**
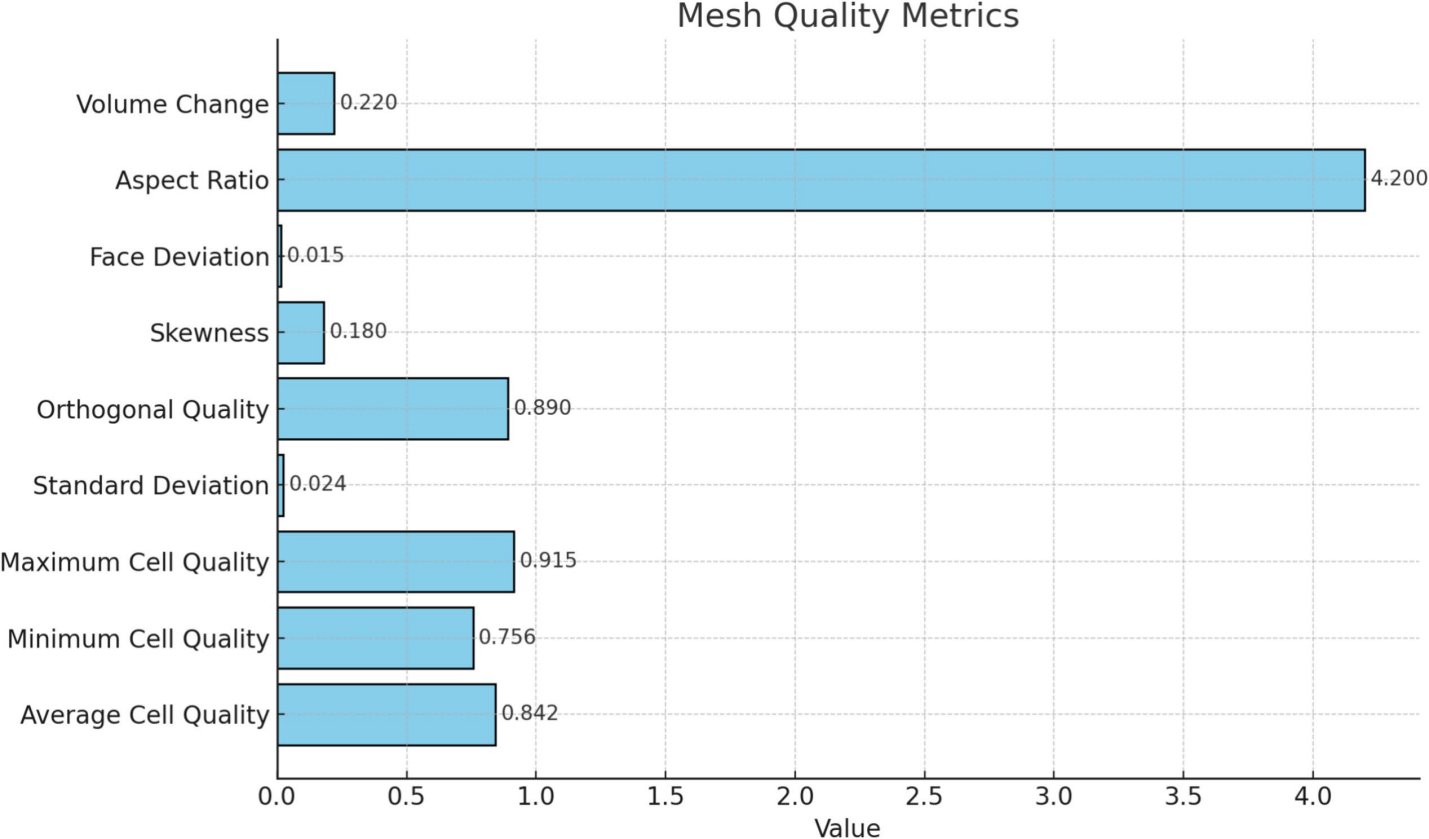
Mesh quality distribution.

The optimized geometric model of the ventilation and dust removal system was imported into mesh generation software for structured meshing. The geometric structure and mesh distribution are shown in Figure 6. The final mesh consisted of 1,265,417 nodes, with an average mesh element quality of 0.842. The mesh quality distribution is illustrated in Figure 7.

From Figure 6, the orthogonal skewness (0.89) and skewness factor (0.18) indicate that the overall shape of the mesh is regular, with minimal geometric distortion, making it suitable for simulating complex phenomena such as flow fields. The surface degradation metric (0.015) demonstrates high surface smoothness, which facilitates the accurate application of boundary conditions. The volumetric change rate of adjacent elements (0.22) is small, ensuring high simulation accuracy in regions with steep gradients. Mesh adjacency (4.2) reflects the acceptable elongation of mesh elements, with no significant distortion observed.

The mesh was imported into the Fluent solver for simulation. The turbulence model was configured as a realizable two-equation model. The inlet boundary was set to “velocity inlet,” the outlet boundary to “pressure outlet,” and all walls were defined as “no-slip solids.” The fluid type was specified as “air.” The Discrete Phase Model (DPM) was enabled, with parameters detailed in Table 2.

**Table 2:** Fluent parameter settings.

Parameter category	Settings
Interaction with continuous phase	On
DPM iteration interval	200
Injection type	Surface
Particle TYPE	Inert
Material	Coal-hv
Diameter distribution	rosin-rammler
Total flow rate/(kg · s^一1^)	0.002 5
Min diameter/μm	1 × 10^−6^
Max diameter/μm	1 × 10^−4^
Mean diameter/μm	1 × 10^−5^
Spread parameter	3.5
Number of diameters	15
Injection velocity/(m·s^−1^)	10
Boundary condition for particles	Reflect
Drag law	Spherical
Turbulence interaction	Enabled
Particle heat transfer	Enabled
Gravity/(m·s^−2^)	9.81

**Figure 8:**
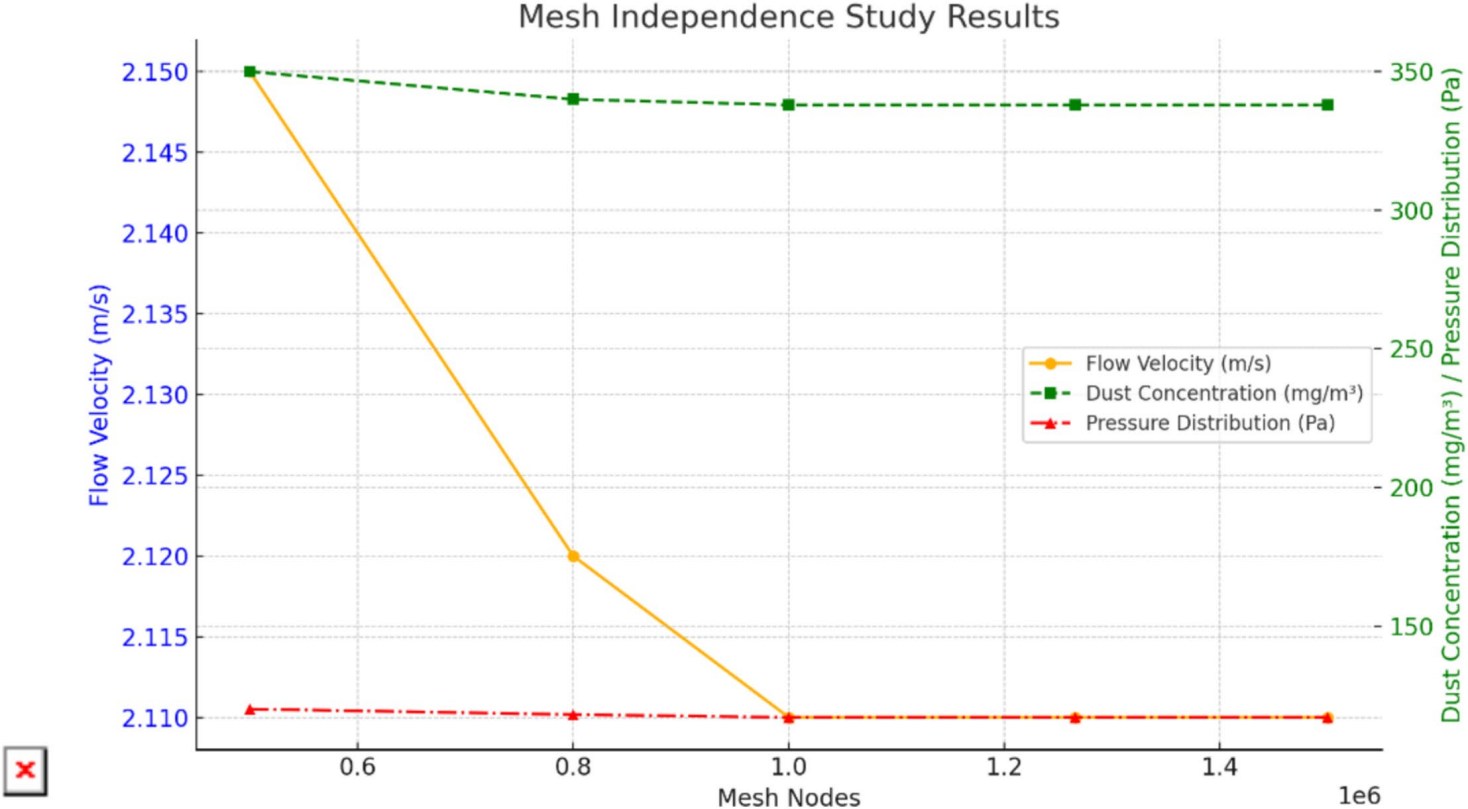
Mesh independence validation.

A mesh independence study was conducted to verify the reliability of the simulation results by testing variations in flow field parameters across different mesh densities from coarse to fine. The key performance indicators analyzed include flow velocity, dust concentration, and pressure distribution, as illustrated in Figure 8. The results demonstrate that, once the mesh node count reaches 1,265,417, further refinement of the mesh has negligible impact on the simulation results, confirming that this mesh density is sufficient to accurately capture the physical phenomena (Hao et al., 2024; Zhang et al., 2021).

From Figure 8, it can be observed that as the mesh node count increases, key performance parameters such as flow velocity, dust concentration, and pressure distribution gradually stabilize, indicating diminishing influence of mesh density on simulation results. Regarding flow velocity, the variation significantly decreases with increasing mesh density. At 1,265,417 nodes, the velocity stabilizes at approximately 2.11 m/s, with further mesh refinement showing minimal impact. Dust concentration exhibits considerable fluctuations at lower mesh densities but becomes increasingly uniform as the mesh density improves, ultimately stabilizing at the current density, accurately reflecting dust distribution patterns. Pressure distribution, on the other hand, shows minimal variations throughout, demonstrating low sensitivity to mesh refinement and achieving stability quickly, even at lower mesh densities. This suggests high computational accuracy for pressure distribution with modest mesh requirements. These findings validate that a mesh density of 1,265,417 nodes is sufficient to provide accurate results, balancing simulation precision and computational efficiency. In the simulation stage, we selected four pressure-to-suction ratios (4:5, 1:1, 4:3, and 2:3), and these selections were based on the analysis of the actual ventilation needs of the mine and the effects of different airflow organizations on dust control efficiency. Each ratio represents a different operating condition of the ventilation system. The primary reason for selecting these ratios was to evaluate the dust removal performance of the system under different airflow configurations and air volumes, and to identify the optimal operating parameters. This approach ensures the reliability and applicability of the simulation results.

### 3.2 CNN-LSTM intelligent dust removal model

#### 3.2.1 Parameter determination

After determining the overall structure of the ventilation and dust removal system and the pressure-to-suction ratio, optimizing key parameters becomes essential for enhancing dust removal efficiency.

Distance Between the Ventilation Duct and Dust-Producing Surface (d): This parameter significantly influences the flow field coverage and suction performance. When the duct is positioned closer to the dust-producing surface, suction efficiency improves but coverage is limited, reducing control over large-scale dust dispersion. Conversely, positioning the duct too far results in a sharp decline in suction efficiency. Therefore, a well-defined distance is critical to balance coverage and suction performance.

Height of the Ventilation Duct from the Ground (H): This determines the primary region of airflow movement and affects the spatial scope of dust removal. When the duct height is lower, the airflow primarily concentrates near the ground, effectively capturing surface dust but potentially disrupting airflow organization above the working surface. Conversely, increasing duct height expands the airflow coverage, aiding in capturing more dispersed dust but reducing control over ground-level dust. Given the complex dust distribution in mining environments, reasonable adjustment of duct height is critical to ensure effective dust removal under diverse operational conditions.

Spacing Between Pressure and Suction Ducts (h): This parameter significantly impacts the generation of the Coandă effect and airflow stability. If the spacing is too small, the airflow attachment ability diminishes, leading to localized turbulence and destabilized flow, reducing dust removal efficiency. Excessive spacing, on the other hand, weakens the wall-attachment capability of the Coandă effect, destabilizing inner vortex flow and impairing overall system performance. Proper spacing optimizes the Coandă effect, enhancing airflow stability and uniformity, and improving the overall performance of the ventilation and dust removal system.

**Figure 9:**
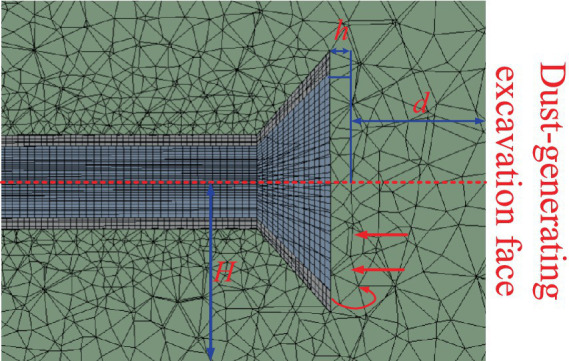
Ventilation and dust removal system control parameters.

Based on this analysis, d, H, and h were selected as the key control parameters for optimization. Coupled with the Coandă effect, these parameters were used to construct a more stable and efficient flow field structure, as depicted in Figure 9 (Brodny and Tutak, 2021). Precise tuning of these parameters enables fine-grained control over airflow characteristics, maximizing the potential of the Coandă effect, and significantly improving the efficiency and reliability of the ventilation and dust removal system.

#### 3.2.2 Dataset collection for control parameters

**Figure 10:**
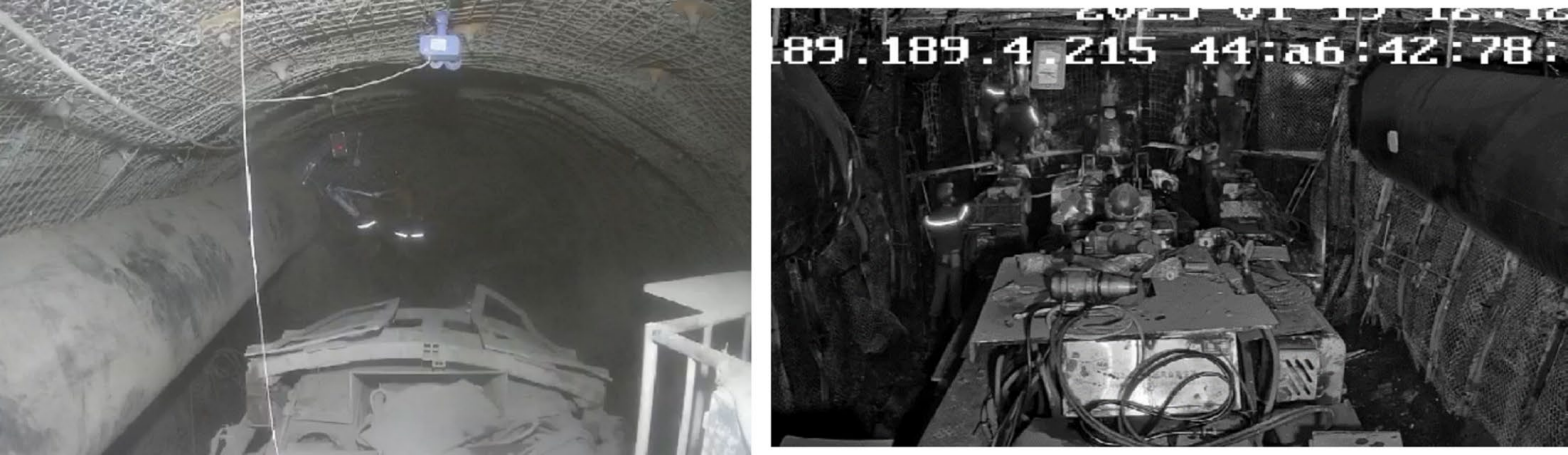
Working face 22,106.

The tunnel dimensions were set as 3.2 m × 1.6 m × 1.6 m (length × width × height). Laser dust detection methods were used to measure the average dust concentration at the roadheader position, and dust concentration measuring instruments were utilized to measure dust concentration at the downwind pedestrian breathing zone, as shown in Figure 10.

Based on the 22,106 working face, the control parameters were set as follows:

Distance between ventilation duct and dust-producing surface (d): Adjustment step size 0.05 m, range 0.3–0.5 m.

Height of the ventilation duct centerline from the ground (H): Adjustment step size 0.05 m, range 0.45–0.55 m.

Spacing between pressure and suction ducts (h): Adjustment step size 0.2 m, range 0–0.2 m.

A full-factorial experiment was conducted, resulting in 45 parameter adjustment schemes, as listed in Table 3.

**Table 3:** Control parameter schemes.

No.	*d*/m	*h*/m	*H*/m
1	0.30	0.2	0.50
2	0.30	0.0	0.50
3	0.30	0.1	0.50
4	0.35	0.2	0.50
5	0.35	0.0	0.50
6	0.35	0.1	0.50
7	0.40	0.2	0.50
8	0.40	0.0	0.50
9	0.40	0.1	0.50
10	0.45	0.2	0.50
11	0.45	0.0	0.50
12	0.45	0.1	0.50
13	0.50	0.2	0.50
14	0.50	0.0	0.50
15	0.50	0.1	0.50
16	0.30	0.2	0.55
17	0.30	0.0	0.55
18	0.30	0.1	0.55
19	0.35	0.2	0.55
20	0.35	0.0	0.55
21	0.35	0.1	0.55
22	0.40	0.2	0.55
23	0.40	0.0	0.55
24	0.40	0.1	0.55
25	0.45	0.2	0.55
26	0.45	0.0	0.55
27	0.45	0.1	0.55
28	0.50	0.2	0.55
29	0.50	0.0	0.55
30	0.50	0.1	0.55
31	0.30	0.2	0.45
32	0.30	0.0	0.45
33	0.30	0.1	0.45
34	0.35	0.2	0.45
35	0.35	0.0	0.45
36	0.35	0.1	0.45
37	0.40	0.2	0.45
38	0.40	0.0	0.45
39	0.40	0.1	0.45
40	0.45	0.2	0.45
41	0.45	0.0	0.45
42	0.45	0.1	0.45
43	0.50	0.2	0.45
44	0.50	0.0	0.45
45	0.50	0.1	0.45

To ensure the reliability and robustness of the model, 25 experiments were conducted for each adjustment scheme under different initial dust concentrations, allowing for a total of 45 adjustment schemes. The dust concentrations were measured at two key locations: the roadheader position and the downwind position. Each experiment took into account varying parameters such as the distance between the ventilation duct and dust-producing surface (d), the height of the ventilation duct (H), and the spacing between the pressure and suction ducts (h), as shown in Table 4. The data summarized in Table 4 illustrates the impact of these control parameters on dust removal efficiency across a range of initial dust concentrations, which varied from relatively low levels to much higher concentrations. At the roadheader position, the dust concentrations showed substantial reductions after the ventilation system was applied. For example, with an initial dust concentration of 547 mg/m^3^, the dust concentration at the roadheader was reduced to 135 mg/m^3^, representing a significant reduction. Similarly, the dust concentration at the downwind position also exhibited notable reductions, confirming the effectiveness of the ventilation system in controlling dust dispersion. In total, the table provides detailed experimental results across all 45 schemes, highlighting how different parameter combinations influence dust concentration levels in the system. These results are critical for understanding the role of each control parameter and for optimizing the system’s performance in real-world applications. The dataset not only helps in evaluating the system’s dust removal capabilities but also lays the foundation for refining the model to improve its predictive accuracy.

**Table 4:** Experimental data on the influence of control parameters on dust removal efficiency.

No.	Initial dust concentration/(mg·m^−3^)		*d*/m	*h*/m	*H*/m	Post-ventilation dust concentration/(mg·m^−3^)	
	Roadheader position	Downwind position				Roadheader position	Downwind position
1	547	521	0.35	0	0.45	135	289
2	384	374	0.35	0.2	0.45	205	352
3	601	521	0.35	0.1	0.45	245	182
4	951	947	0.40	0.2	0.45	290	300
5	857	978	0.40	0	0.45	247	225
:	:	:	:	:	:	:	:
1,121	678	654	0.35	0	0.55	183	125
1,122	548	571	0.35	0.1	0.55	197	168
1,123	228	231	0.30	0.2	0.55	170	255
1,124	471	482	0.30	0	0.55	235	179
1,125	412	401	0.30	0.1	0.55	280	290

## 4 Experiment and simulation results analysis

### 4.1 Numerical simulation analysis

#### 4.1.1 Determination of the optimal pressure-to-suction ratio

**Figure 11:**
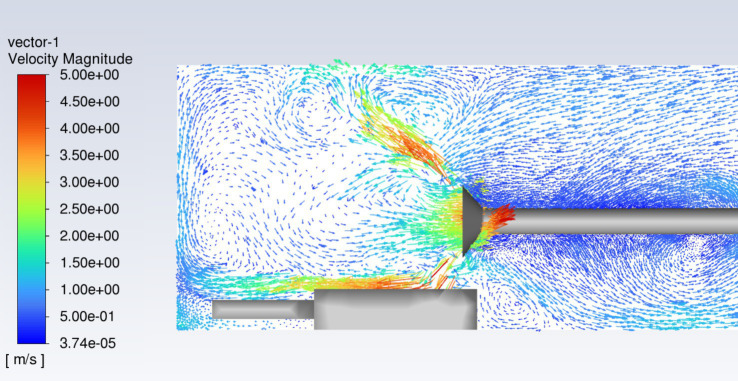
Airflow at the suction pipe opening.

The dust removal efficiency of the ventilation and dust removal system is primarily influenced by the airflow field within the tunnel. By altering the pressure-to-suction ratio, the airflow direction within the field can be controlled, leading to different dust movement patterns and, consequently, varying dust removal efficiencies. For the simulated airflow field matching the dimensions of the simulated tunnel, the supply airflow rate of the suction duct was fixed at 240 m^3^/min. When the pressure-to-suction ratio was set to 1:1, and the improved pressure-suction ducts were employed, a rotational airflow wrapping inward was generated at the duct opening, thereby expanding the dust removal area. The airflow distribution at the suction duct opening is illustrated in Figure 11.

**Figure 12:**
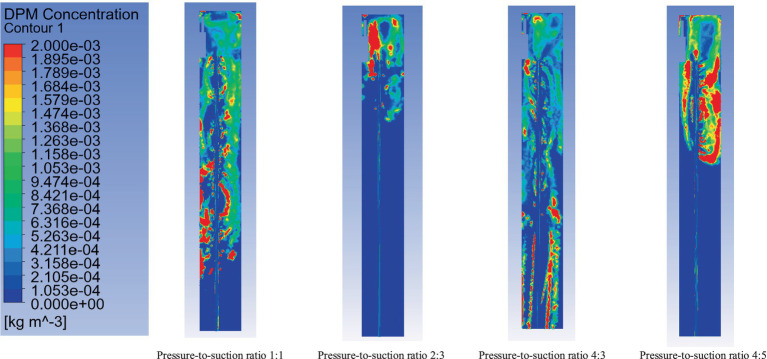
Dust distribution at cross-section x = 2.5 m.

**Figure 13:**
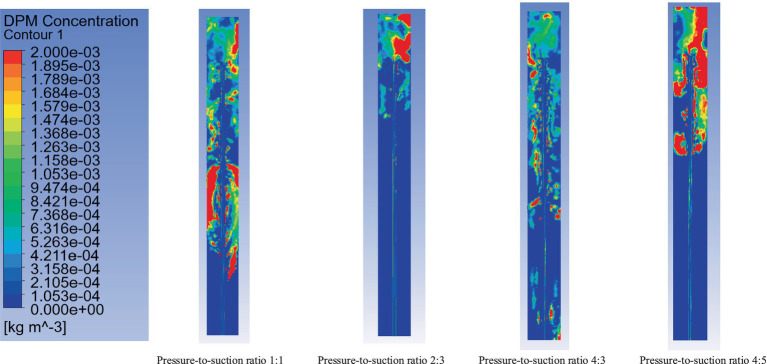
Dust distribution at cross-section y = 2.0 m.

**Figure 14:**
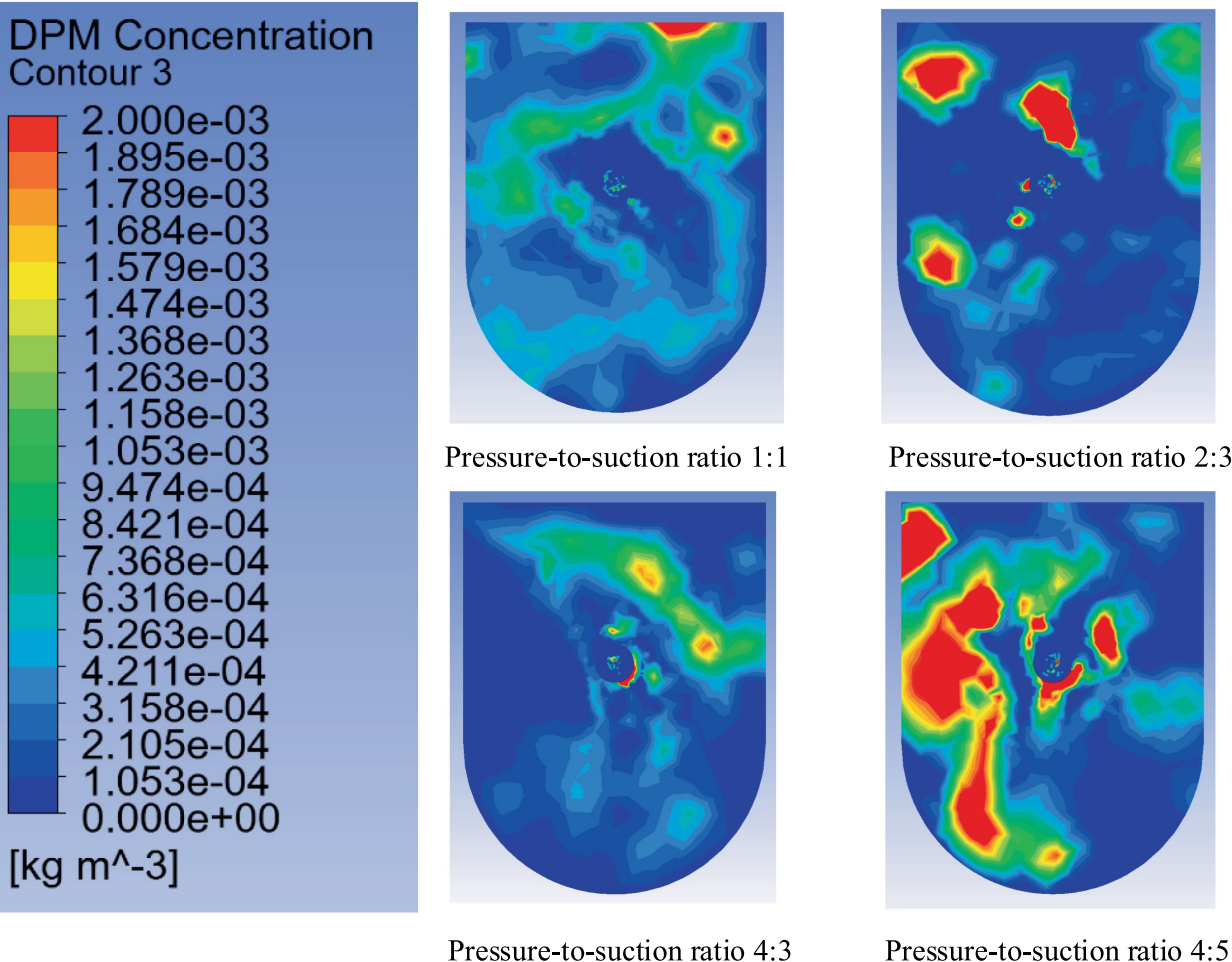
Dust distribution at cross-section z = 7.0 m.

By fixing the suction airflow at 240 m^3^/min, pressure-to-suction ratios of 4:5, 1:1, 4:3, and 2:3 were simulated. The simulation analyzed the dust concentration distribution at the roadheader position (coordinates: y = 2.0 m, x = 2.5 m, z = 7 m), as shown in Figures 12–14 (time-weighted average). The key parameters such as flow velocity and dust concentration were measured at specific locations within the ventilation system. Flow velocity measurements were taken at critical points, including the inlet, outlet, and key positions along the tunnel. Dust concentration was measured at two critical locations: at the roadheader position and at the downwind pedestrian breathing zone. These locations were selected to capture the full range of airflow and dust dispersion, ensuring a comprehensive analysis of the system’s performance. These measurements are vital for validating the effectiveness of the optimized ventilation and dust removal system.

Figure 11 illustrates the airflow distribution at the suction pipe opening, showing the characteristics of the turbulent regions and velocity distribution. The velocity magnitude is represented by the color legend, with red areas indicating high velocity (close to 5 m/s) and blue areas representing low velocity (near zero). It can be observed that a distinct high-velocity region forms near the suction pipe opening, while farther from the opening, the velocity gradually decreases, forming a low-speed flow area. In the high-speed flow region near the suction pipe opening, the airflow is significantly drawn in, with concentrated flow direction and dense streamlines, indicating a strong suction effect.

Figure 12 shows the dust concentration distribution at cross-section x = 2.5 m under different pressure-to-suction ratios. The dust concentration variation is analyzed qualitatively using the color legend to interpret the impact of different pressure-to-suction ratios on dust distribution.

Pressure-to-suction ratio 1:1: Under this condition, the dust concentration distribution exhibits a large high-concentration area, especially near the roadheader. The red and orange regions indicate high dust concentrations, suggesting insufficient airflow to effectively control dust diffusion. In this case, the airflow organization is unstable, making it difficult to form an effective dust collection effect.

Pressure-to-suction ratio 2:3: When the ratio is adjusted to 2:3, the high-concentration dust areas are significantly reduced, and the dust concentration distribution becomes more uniform. Dust is effectively directed into the suction pipe, indicating that the airflow organization under this ratio is more reasonable, effectively controlling dust diffusion and improving dust removal efficiency.

Pressure-to-suction ratio 4:2: Under the ratio of 4:2, the high-concentration dust areas increase again, particularly near the roadheader, with more red and orange regions. This suggests that while the pressure airflow is larger, it fails to form a synergistic effect with the suction, leading to an increased dust diffusion range.

Pressure-to-suction ratio 4:5: With this ratio, the dust diffusion range is large, and the high-concentration regions are more concentrated. Although the suction airflow increases, the airflow organization may be disrupted, failing to effectively remove dust and resulting in significant dust residue.

Figure 13 illustrates the dust distribution at cross-section y = 2.0 m under different pressure-to-suction ratios, revealing the variation patterns and effects of dust concentration with changes in the ratio.

Pressure-to-suction ratio 1:1: Under this ratio, the figure shows a wide range of dust concentration distribution, particularly near the roadheader, with noticeable high-concentration dust (red regions occupying a large proportion). This indicates poor airflow organization under this ratio, making it difficult to effectively control dust diffusion and resulting in dust accumulation over a large spatial area.

Pressure-to-suction ratio 2:3: When the ratio is adjusted to 2:3, the high-concentration dust regions are significantly reduced, and the dust concentration distribution becomes more uniform. Most areas show reduced dust concentration to green or blue levels. This demonstrates that this ratio optimizes airflow organization, effectively guiding dust to the suction pipe and improving dust removal efficiency.

Pressure-to-suction ratio 4:2: Under the ratio of 4:2, the high-concentration dust regions expand, especially near the roadheader and downstream areas, with more red and orange regions. This may result from excessive pressure airflow causing turbulence and exacerbating dust diffusion.

Pressure-to-suction ratio 4:5: With a ratio of 4:5, the dust distribution characteristics are similar to those under the ratio of 1:1. Although the suction airflow increases, the overall high-concentration regions remain large, and the dust diffusion range increases, indicating that this ratio does not effectively improve airflow organization or dust removal efficiency.

Figure 14 shows the dust concentration distribution at cross-section z = 7.0 m under different pressure-to-suction ratios.

Pressure-to-suction ratio 1:1: Under this ratio, the overall dust concentration is relatively low, with the cross-section primarily showing blue regions and a small number of green areas near the center. This indicates relatively uniform airflow organization under this ratio, with a smaller dust diffusion range. However, it does not completely concentrate the dust towards the suction pipe.

Pressure-to-suction ratio 2:3: When the ratio is adjusted to 2:3, the dust concentration distribution within the cross-section becomes more concentrated. The red and orange regions are significantly reduced, and the dust is mainly concentrated near the center, with the overall concentration decreasing. This demonstrates that this ratio optimizes airflow paths, guiding more dust towards the suction pipe and enhancing dust removal efficiency.

Pressure-to-suction ratio 4:3: Under the ratio of 4:3, the high-concentration dust regions slightly expand, with an increase in green and yellow areas. This indicates that excessive pressure airflow may cause further dust diffusion within the cross-section, reducing the suction pipe’s dust control efficiency.

Pressure-to-suction ratio 4:5: When the ratio is 4:5, the high-concentration dust regions within the cross-section expand significantly, with large areas of red and orange regions appearing. This suggests that excessive suction airflow leads to turbulence, causing more severe dust diffusion within the cross-section, making it difficult to concentrate dust effectively and reducing dust removal efficiency.

From the analysis of Figures 11–13, when the inflow rate is less than the outflow rate (pressure-to-suction ratios of 4:5 and 1:1), insufficient inflow leads to substantial dust accumulation at the front-end of the working face near the roadheader and operator. When the inflow rate exceeds the outflow rate (pressure-to-suction ratios of 4:3 and 2:3), sufficient inflow can scatter the dust accumulating at the working face and direct it into the suction pipe. When the pressure-to-suction ratio is 2:3, the dust concentration distribution at cross-sections x = 2.5 m and y = 2.0 m is optimal. However, under pressure-to-suction ratios of 4:5 and 1:1, the dust concentration at cross-section z = 7.0 m is extremely high, while the ratio of 2:3 achieves the lowest dust concentration at this cross-section. In conclusion, the optimal pressure-to-suction ratio for the ventilation and dust removal system is 2:3.

### 4.2 Intelligent control of ventilation and dust removal system parameters based on CNN-LSTM

To ensure the reliability and accuracy of the CNN-LSTM model in predicting dust concentration, a rigorous parameter selection process was implemented. The selection of key hyperparameters was guided by both empirical testing and optimization techniques:

Kernel size and number of filters in CNN layer: The CNN component extracts spatial features from the input data, and its kernel size was optimized to (2,1), ensuring a balance between feature resolution and computational efficiency. The number of filters was set to 64 to capture sufficient spatial correlations.

LSTM hidden units: The LSTM layer was used to process time-series dependencies in dust concentration data. Based on multiple trials, 64 hidden units were chosen to provide a trade-off between model complexity and training efficiency (Lu et al., 2023).

Activation function and optimizer: The rectified linear unit (ReLU) activation function was used in convolutional layers, while the Adam optimizer was selected for efficient gradient updates.

Training-validation split: The dataset was split into 80% for training and 20% for testing to ensure generalization.

For real-time application, the trained CNN-LSTM model was integrated into the ventilation control system. It receives continuous real-time input of environmental parameters, including airflow velocity, duct position, and initial dust concentration. Based on the predicted dust distribution patterns, the system automatically adjusts key parameters such as the duct positioning to optimize dust removal efficiency. This adaptive control mechanism ensures that the system can dynamically respond to varying operational conditions, reducing energy consumption while maintaining high dust removal efficiency.

#### 4.2.1 Training and performance comparison of CNN-LSTM model

To achieve intelligent control of the parameters in the ventilation and dust removal system, the CNN-LSTM model was adopted to predict and optimize the system’s operational parameters. The CNN module was designed with a single convolutional layer, where the convolution kernel size was set to (2, 1), and the number of kernels was 64, ensuring the extraction of multi-level feature information. After feature extraction by the convolutional layer, the extracted feature sequence was input into the LSTM module for time series modeling and capturing long-term dependencies. The LSTM module was implemented with a two-layer stacked structure, each layer containing 64 units. Through a gating mechanism, it effectively avoided gradient vanishing and explosion issues commonly found in long-term dependency problems, thereby improving the model’s dynamic response capability and prediction accuracy in controlling ventilation and dust removal parameters. After processing in the LSTM module, the feature sequence was fed into a fully connected layer with 64 neurons for regression prediction, where the activation function used in the fully connected layer was ReLU. The CNN-LSTM hybrid model was employed for predicting dust concentrations in the ventilation and dust removal system. The model’s input features include the control parameters of the ventilation system, such as the distance between the duct and the dust-producing surface (d), the height of the ventilation duct (H), and the spacing between the pressure and suction ducts (h). Additionally, initial dust concentration values and other environmental variables were used to train the model. The model predicts dust concentrations at the roadheader and downwind positions, which are the key performance indicators that we aim to optimize. The CNN-LSTM hybrid model consists of CNN layers for spatial feature extraction and LSTM layers to capture temporal dependencies in the data. The model was trained using the Adam optimization algorithm, with 80% of the dataset used for training and the remaining 20% used for testing. The model’s performance was evaluated using metrics such as Mean Absolute Error (MAE), Root Mean Squared Error (RMSE), and the coefficient of determination (R^2^). To ensure the accuracy and consistency of the dataset, the training data was standardized to ensure all features were on the same scale, aiding the model’s convergence. Missing or inconsistent data points were addressed using interpolation or imputation techniques. For model validation, the evaluation metrics MAE, RMSE, and R^2^ were utilized to assess prediction accuracy. The results demonstrated that the CNN-LSTM model outperformed the baseline BP neural network model in predicting dust concentrations. Furthermore, the predicted dust concentrations were compared with actual measurements, confirming the model’s accuracy and reliability. To verify the superiority of the CNN-LSTM model, the BP neural network was chosen as a benchmark model for comparison. The BP network architecture was configured as 5–13 − 2, meaning it contained 5 nodes in the input layer, 13 nodes in the hidden layer, and 2 nodes in the output layer. The hidden layer utilized the tanh activation function. During model training, both the CNN-LSTM model and the BP neural network employed the Adam optimization algorithm, with the number of training steps set to 1,000. The training dataset accounted for 80% of the total samples, while the remaining 20% was used for testing.

**Figure 15:**
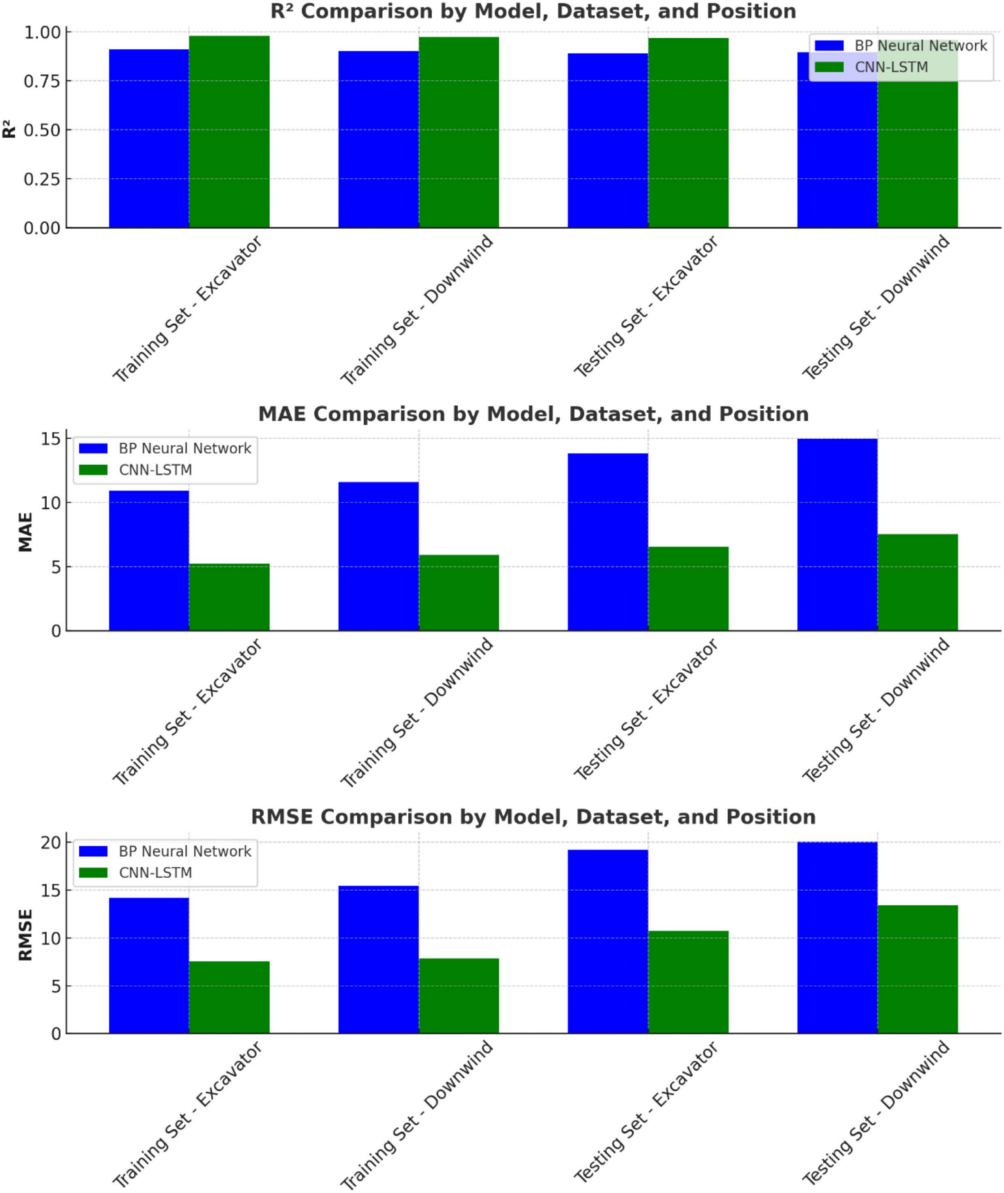
Performance comparison between bp neural network and CNN-LSTM model.

After training was completed, the performance of the CNN-LSTM model was compared with that of the BP neural network. The results are shown in Figure 15. The comparative analysis indicates that the CNN-LSTM model, by extracting spatial features via convolution operations and modeling time dependencies with LSTM, demonstrated significantly higher accuracy and stability in parameter prediction for the ventilation and dust removal system. This robust performance highlights the technical support provided by the CNN-LSTM model for the intelligent control of system parameters.

As shown in Figure 15, the CNN-LSTM model demonstrates significantly superior performance in predicting parameters for the ventilation and dust removal system compared to the BP neural network. Specifically, for predictions at the roadheader, the R^2^ values of the CNN-LSTM model for the training and testing datasets were 0.9783 and 0.9752, respectively, compared to 0.9124 and 0.9018 for the BP neural network. This indicates that the CNN-LSTM model effectively captures nonlinear relationships in the data and achieves higher fitting accuracy.

The MAE and RMSE values for CNN-LSTM at the roadheader were also significantly lower. For the training dataset, the MAE decreased from 10.8965 to 5.2347, and the RMSE decreased from 14.2053 to 7.5346. For the testing dataset, the MAE and RMSE decreased from 11.5642 and 15.4267 to 5.8972 and 7.8659, respectively. These improvements highlight the enhanced precision and stability of the CNN-LSTM model.

At the downwind side, the CNN-LSTM model again showed superior performance, with R^2^ values of 0.9682 and 0.9594 for the training and testing datasets, compared to 0.8923 and 0.8951 for the BP neural network. Similarly, the MAE and RMSE decreased significantly, with the training dataset MAE reducing from 13.7842 to 6.5428 and RMSE reducing from 19.2346 to 10.7623. The testing dataset saw reductions in MAE from 14.9278 to 7.5236 and RMSE from 20.0425 to 13.4271. These results demonstrate the CNN-LSTM model’s superior ability to capture long-term temporal dependencies and the dynamic variations of complex features in time-series data.

#### 4.2.2 Validation of CNN-LSTM model accuracy

To validate the accuracy of the CNN-LSTM model in predicting dust concentration, 15 parameter control schemes were selected from the optimized parameter experiment platform. A comparative analysis was conducted between the measured data and the model’s predicted results.

**Figure 16:**
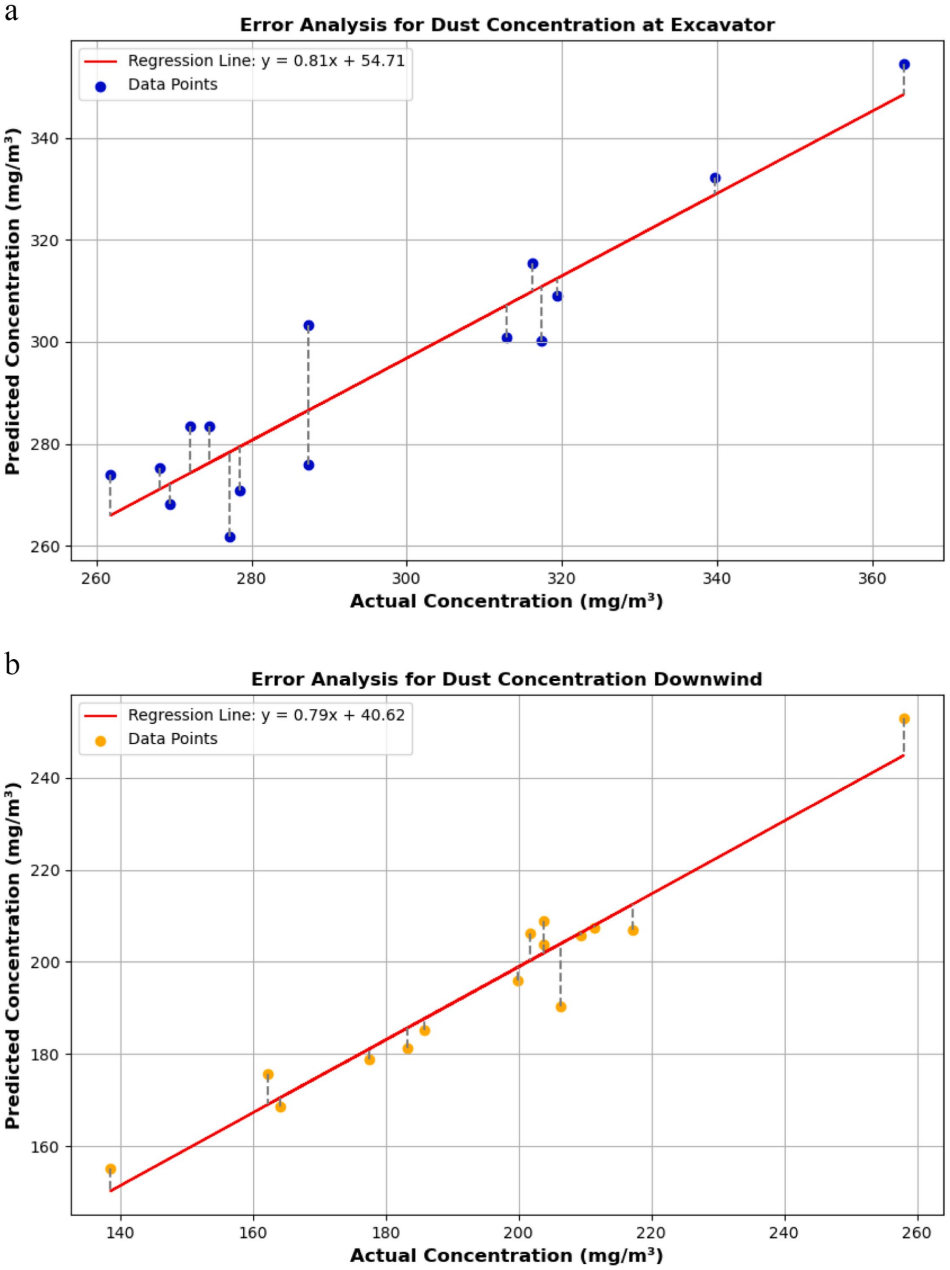
Model prediction vs. actual values. **(a)** Roadheader position. **(b)** Downwind position.

At the roadheader, the error range between the actual and predicted dust concentrations was 0.39–6.01%. The predicted results exhibited high consistency with the actual values. Figure 16A shows that the data points closely align with the regression line, indicating the model’s strong responsiveness to changes in dust concentration at the roadheader. This accuracy can be attributed to the stability of the ventilation and dust removal system parameters and the enhancement of airflow optimization through the Coandă effect.

For the downwind side, the prediction error range was 0.05–9.15%. Figure 16B shows that while most data points are near the regression line, some deviations were observed, potentially due to turbulence and dust diffusion uncertainties under actual working conditions. Nevertheless, the model maintained high prediction accuracy under complex flow field conditions.

In summary, the prediction errors for both the roadheader and the downwind side were minimal. The CNN-LSTM model effectively captured the nonlinear relationships between key parameter changes and dust concentration distributions. Combined with the optimized long-pressure, short-suction ventilation and dust removal system based on the Coandă effect, the model exhibited excellent performance. It provides robust technical support for intelligent system regulation, showcasing high prediction accuracy and generalization capability in practical engineering applications.

#### 4.2.3 Optimization of ventilation and dust removal system parameters

**Figure 17:**
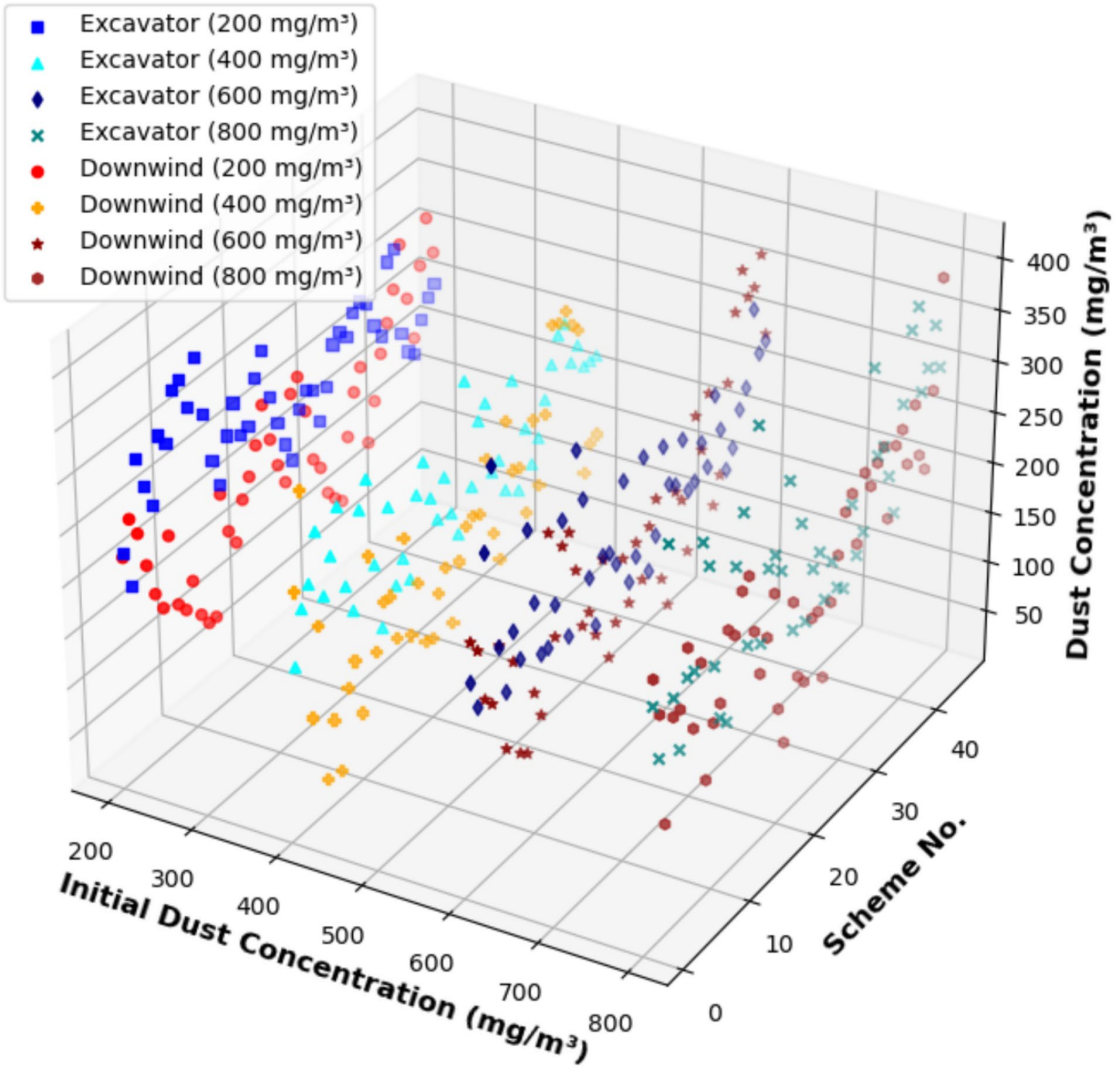
Predicted results of dust concentration under different initial dust concentrations.

The initial dust concentrations were set to 200, 400, 600, and 800 mg/m^3^, and the trained CNN model was used to predict the dust concentrations after applying 45 parameter control schemes. The results are shown in Figure 17. The analysis indicates that higher initial dust concentrations result in higher dust concentrations after removal, demonstrating a significant impact of the initial concentration on the overall dust removal efficiency. Dust concentration at the downwind side was consistently higher than at the roadheader, particularly at higher initial concentrations, likely due to dust accumulation downstream in the airflow.

For an initial dust concentration of 200 mg/m^3^, parameter control schemes 15, 20, and 32 achieved ideal dust removal effects with relatively low dust concentrations, while schemes 10 and 11 resulted in relatively high dust concentrations. At an initial dust concentration of 400 mg/m^3^, schemes 11 and 27 provided better dust removal efficiency. This demonstrates that the optimal parameter control combinations vary under different initial dust concentrations, necessitating appropriate control schemes tailored to specific working conditions to achieve optimal dust removal efficiency.

The optimal parameter control schemes and their respective dust removal efficiencies for initial dust concentrations ranging from 200 to 800 mg/m^3^ at both the roadheader and downwind side are presented in Table 5.

**Table 5:** Dust removal efficiency of optimal control schemes under different initial dust concentrations.

Initial dust concentration/(mg·m^−3^)	Optimal control scheme	*d*/m	*h*/m	*H*/m	Dust concentration at roadhead/(mg·m^−3^)	Dust concentration at downwind side/(mg·m^−3^)	Average dust removal efficiency/%
200	30	0.50	0	0.55	150.25	128.67	52.13
400	12	0.45	0	0.50	95.43	125.12	76.85
600	22	0.40	0.2	0.55	158.67	90.34	80.24
800	8	0.40	0.0	0.50	170.78	110.56	84.12

From Table 5, it can be observed that for an initial dust concentration of 200 mg/m^3^, scheme 30 achieved the best dust removal effect, reducing the dust concentration at the roadheader by 49.88% and at the downwind side by 55.67%. For an initial dust concentration of 800 mg/m^3^, scheme 8 provided the optimal effect, reducing the dust concentration at the roadheader by 78.90% and at the downwind side by 86.18%. The reasonable optimization of control parameters significantly improves the performance of the ventilation and dust removal system. Particularly at high initial dust concentrations, the optimized schemes achieve a more substantial reduction in dust concentration, fully validating the effectiveness and applicability of the optimized control schemes.

Several potential error sources were identified and analyzed:

Environmental Variability: Despite efforts to maintain stable ventilation conditions, minor fluctuations in airflow due to external factors such as machinery operation and worker movement may have influenced dust concentration readings.

Data Processing Errors: Data collection and processing involved multiple steps, and while outliers were removed systematically, minor inconsistencies in recorded values could still affect statistical results.

## 5 Conclusion

### 5.1 Performance of the optimized ventilation system

The optimized system based on the Coandă effect significantly reduces dust dispersion and enhances energy efficiency. By employing a nested duct structure and leveraging the Coandă effect, airflow is directed more effectively along the tunnel walls, minimizing dust concentration in key locations. The system achieves optimal performance under a pressure-to-suction ratio of 2:3, resulting in dust concentration reductions of up to 78.90% at the mining machine location and 86.18% downstream when the initial dust concentration is 800 mg/m^3^.

### 5.2 Intelligent parameter control with CNN-LSTM

The integration of a CNN-LSTM hybrid model for intelligent parameter adjustment demonstrates superior accuracy and adaptability compared to traditional models. The CNN-LSTM model outperforms BP neural networks in predictive accuracy, achieving an R^2^ of 0.9783 at the mining machine and 0.9682 downstream. This intelligent prediction framework enables real-time system optimization by adjusting ventilation system parameters, improving the overall efficiency and adaptability of the ventilation and dust control system under varying working conditions. The model’s predictions have been instrumental in guiding the real-time optimization of key system parameters, ensuring enhanced dust removal performance while reducing energy consumption.

### 5.3 Implications for energy efficiency and safety

The optimized long-pressure short-suction system enhances energy efficiency by eliminating the need for high-power fans, achieving high dust removal efficiency while reducing power consumption. Furthermore, the system’s adaptability to dynamic working conditions ensures enhanced worker safety and operational efficiency. The findings validate the broad applicability of the system across various mining conditions, providing a robust foundation for future advancements in intelligent ventilation and dust control solutions.

### 5.4 Limitations and future work

The optimized long-pressure-short-suction ventilation and dust removal system based on the Coandă effect demonstrates significant advantages in dust suppression efficiency and energy savings. However, its applicability may vary depending on different mining conditions, requiring specific adjustments for optimal performance. The system was designed and validated for a fully mechanized mining face with standard tunnel dimensions. In environments with narrower or wider tunnels, the airflow characteristics and dust diffusion patterns may differ, necessitating adjustments to the spacing between the air supply and exhaust ducts, as well as the pressure-to-suction ratio, to maintain optimal dust removal efficiency. In deeper mining operations or complex underground structures, ventilation resistance tends to increase due to longer airflow paths and potential obstructions, which may affect the effectiveness of the optimized design. To adapt to such conditions, the pressure distribution of the air supply system should be dynamically adjusted to ensure effective airflow attachment along the tunnel surfaces.

Furthermore, while the study primarily focused on the suppression of fine dust particles commonly found in coal mining, different mining environments—such as metal ore or hard rock mining—may present dust particle sizes and compositions that differ significantly, which could impact system efficiency. Therefore, further testing and modifications to the Coandă effect-based duct design may be required to ensure optimal dust capture and removal for varying dust types. Additionally, some underground mining environments may impose specific constraints, such as low clearance heights, limited space for ventilation infrastructure, or varying ambient humidity levels, all of which may influence the airflow attachment effect and dust transport efficiency. Computational simulations and field tests under these specific working conditions would be helpful to refine the system’s adaptability for different mining applications.

Overall, while the proposed ventilation and dust removal system has demonstrated robust performance in standard mining environments, targeted modifications may be necessary when applied to specific underground conditions. Future research will focus on expanding the model’s adaptability to a wider range of mining conditions through field validation and real-time optimization techniques. Despite the promising results, the study has some limitations. The numerical simulations and experimental validations were conducted under specific mining conditions, and further research is needed to evaluate the system’s adaptability to different tunnel geometries and dust concentration levels. Additionally, future work could explore real-time adaptive control strategies to further improve system responsiveness under fluctuating environmental conditions.

## Data Availability

The original contributions presented in the study are included in the article/supplementary material, further inquiries can be directed to the corresponding author.
